# From urge to action: an integrative neuropsychological model of tic generation in Tourette syndrome

**DOI:** 10.3389/fpsyt.2026.1841663

**Published:** 2026-05-25

**Authors:** Lorenzo Zamboni, Angela Federico, Rebecca Casari, Simone Campagnari, Anna Bertoldi, Francesco Righetti, Silvio Masin, Riccardo Pavan, Michele Resina, Fabio Lugoboni

**Affiliations:** 1 Addiction Medicine Unit, Internal Medicine, G.B. Rossi Hospital, Verona, Italy; 2 University of Verona, Verona, Italy; 3 Divergens, Private Healthcare Provision for Neurodivergence, Verona, Italy; 4 Internal Medicine, G.B. Rossi Hospital, Verona, Italy

**Keywords:** inhibitory control, neurodivergence, premonitory urges, tic generation, Tourette syndrome

## Abstract

**Background:**

Tourette syndrome (TS) has traditionally been conceptualized as a movement disorder characterized by involuntary motor and vocal tics. However, growing evidence suggests that tic generation involves complex interactions between neural, physiological, and psychological processes, particularly the role of premonitory urges (PU).

**Objective:**

This paper aims to develop an integrative model of tic generation by synthesizing recent evidence across neurobiological, neurophysiological, and psychological domains.

**Methods:**

A narrative review of PubMed-indexed literature (2020–2026) was conducted, complemented by landmark earlier studies. Evidence was selected and synthesized to explore the relationships between cortico–striato–thalamo–cortical dysfunction, motor inhibition, interoception, and reinforcement mechanisms.

**Results:**

Findings indicate that TS is associated with multilevel dysfunction, including impaired inhibitory control, altered cortical excitability, and abnormal interoceptive processing. Premonitory urges emerge as central drivers of tic behavior, linking internal states to motor output. Tic execution produces temporary relief, reinforcing behavior through negative reinforcement mechanisms. These processes form a dynamic urge–action–relief loop, further shaped by perception–action coupling and neural noise.

**Conclusions:**

TS can be conceptualized as a disorder of action regulation, in which tics arise from the interaction between dysfunctional neural systems, abnormal interoceptive signals, and reinforcement-based learning processes. The proposed model provides a unified and testable framework for understanding tic generation and has potential implications for clinical intervention targeting the functional relationship between urges and behavior.

## 1 Introduction

Tourette syndrome (TS) is a neurodevelopmental disorder characterized by the presence of multiple motor and at least one vocal tic, with onset in childhood and a typically fluctuating course over time (1). Although traditionally conceptualized as a movement disorder, growing evidence indicates that TS reflects dysfunction within distributed neural systems integrating motor, cognitive, emotional, and sensory processes (2, 3).

From a neurobiological standpoint, TS has been consistently associated with alterations in cortico–striato–thalamo–cortical (CSTC) circuits, which play a fundamental role in motor control, action selection, and behavioral inhibition (4). Dysregulation within these circuits, particularly involving the basal ganglia and their cortical projections, is thought to impair inhibitory control and lead to the inappropriate release of motor patterns, clinically manifesting as tics (5).

In addition to circuit-level dysfunction, TS is increasingly understood as involving a complex imbalance across multiple neurotransmitter systems. While dopaminergic dysregulation has historically been central to explanatory models—supported by the clinical efficacy of dopamine receptor antagonists, other perspectives highlight the role of altered GABAergic inhibition and excitatory–inhibitory imbalance in shaping abnormal motor output (6, 7).

Beyond motor manifestations, one of the most distinctive features of TS is the presence of premonitory urges (PU) subjectively aversive sensory or cognitive experiences that precede tic execution and are temporarily relieved by it. These urges are reported by the majority of adolescents and adults with TS and are considered a core component of the disorder’s phenomenology (8–10). Neuroimaging studies suggest that premonitory urges are associated with activity in brain regions involved in interoception and motor preparation, including the insula and supplementary motor area (SMA) (11, 12).

The close temporal and functional relationship between premonitory urges and tics has led to a reconceptualization of tic generation as an urge-driven process, rather than a purely involuntary motor phenomenon. In this framework, tics can be understood as actions performed to relieve internal discomfort, thus sharing features with compulsive behaviors characterized by urge–action–relief cycles (13). Behavioral evidence further supports this view, showing that tic suppression is possible but effortful and typically associated with an increase in internal tension, suggesting partial voluntary control modulated by top-down inhibitory mechanisms (14, 15).

Neurophysiological studies have also contributed to this evolving understanding, demonstrating abnormalities in motor cortex excitability and inhibitory control in individuals with TS. In particular, reduced intracortical inhibition has been consistently observed, supporting the hypothesis of impaired GABAergic inhibitory mechanisms (16). These findings reinforce the idea that tics emerge from dysfunctional interactions between sensory input, internal states, and motor output systems.

Despite these advances, current models of TS remain fragmented, with neurobiological, neurophysiological, and psychological findings often addressed separately rather than integrated into a unified framework. In particular, the functional role of premonitory urges within the broader pathophysiology of TS remains insufficiently conceptualized.

In this context, there is a need for a comprehensive integrative model that bridges these domains. The present paper aims to address this gap by synthesizing current evidence and proposing a framework in which tics are conceptualized as regulatory behaviors emerging from dysfunctional neural systems and maintained through reinforcement mechanisms linked to the relief of premonitory urges.

## 2 Methods

### 2.1 Study design

This paper was designed as a narrative, concept-oriented review aimed at integrating current evidence on the psychological and neurophysiological function of tics in Tourette syndrome (TS), rather than quantitatively synthesizing treatment effects or estimating pooled effect sizes. A narrative review design was chosen because the topic spans heterogeneous domains, including phenomenology, neurobiology, neurophysiology, interoception, inhibition, and reinforcement mechanisms, which are not easily reducible to a single outcome framework. This approach is consistent with methodological recommendations emphasizing that narrative reviews are particularly useful for interpretative integration and theory development (17, 18).

### 2.2 Search strategy

A structured literature search was conducted in PubMed only, in line with the scope of the present project. The search focused on publications indexed from January 1, 2020, to March 22, 2026, in order to capture the most recent evidence from the last five years, while allowing inclusion of early online articles indexed in PubMed.

Additional landmark papers published before 2020 were included when necessary to contextualize key constructs such as premonitory urges, inhibitory control, and sensorimotor integration.

Landmark papers were defined as highly influential studies that substantially contributed to the conceptualization of core mechanisms relevant to the present framework, including premonitory urges, inhibitory control, sensorimotor integration, and reinforcement processes in Tourette syndrome. Selection of these studies was based on their historical relevance, frequent citation within the field, and their recurring inclusion in major reviews and theoretical models of tic generation.

The PubMed search was performed using combinations of Medical Subject Headings and free-text terms related to Tourette syndrome and tic function. The main search strategy included the following conceptual domains:

(“Tourette syndrome” OR “tic disorders” OR tics) AND (“premonitory urge” OR urge) AND (“neurophysiology” OR “sensorimotor” OR “inhibitory control” OR “interoception” OR “reinforcement” OR “habit learning” OR “psychological function”).

To improve sensitivity, additional targeted searches were conducted using narrower strings (e.g., “Tourette syndrome AND premonitory urge review”; “tics AND reinforcement learning”).

### 2.3 Eligibility criteria

Articles were considered eligible if they met the following criteria:

1. published in PubMed-indexed journals;
2. written in English;
3. focused on Tourette syndrome or chronic tic disorders;
4. addressed at least one of the following domains:
  - psychological meaning or function of tics,
  - premonitory urges,
  - neurophysiological correlates of tic generation or suppression,
  - sensorimotor processing,
  - inhibitory control,
  - reinforcement or habit mechanisms;
5. consisted of reviews, systematic reviews, meta-analyses, or original studies relevant to conceptual integration.

Exclusion criteria were:

- studies primarily focused on treatment efficacy without mechanistic discussion;
- studies focused exclusively on genetics or pharmacology without relevance to tic function;
- case reports with limited theoretical contribution;
- non-English publications.

### 2.4 Study selection and data extraction

Titles and abstracts were screened for relevance, followed by full-text evaluation. For each included article, the following information was extracted: publication year, article type, conceptual focus, neurobiological or neurophysiological mechanisms, role of premonitory urges, and implications for tic function.

Because this is a narrative review, no formal risk-of-bias tool was applied. However, preference was given to recent reviews, high-quality synthesis papers, and original studies directly informing the proposed model. This approach is consistent with recommendations for transparent narrative reviews (17).

**Figure 1:**
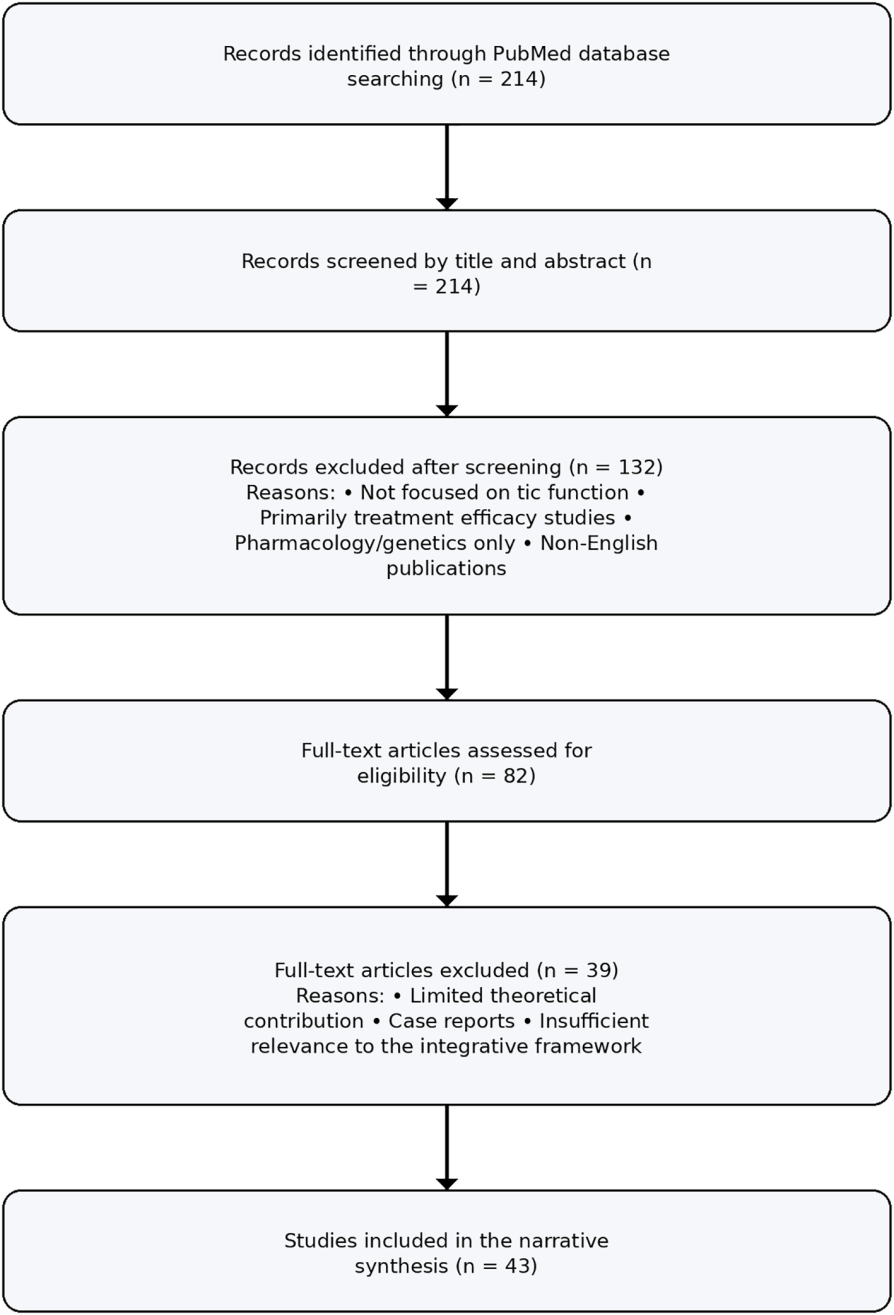
Literature search and study selection process. Flowchart illustrating the identification, screening, eligibility assessment, and inclusion of studies used in the present narrative review. The selection process included PubMed-indexed studies focusing on tic function, premonitory urges, neurophysiology, inhibitory control, and reinforcement mechanisms in Tourette syndrome.

The literature search and study selection process are summarized in **Figure 1** and key studies included in the narrative synthesis are summarized in **Table 1**.

**Table 1:** Summary of key studies included in the narrative synthesis.

Authors	Year	Study type	Main focus	Contribution to the integrative model
Albin & Mink (4)	2006	Review	CSTC circuits and basal ganglia dysfunction	Neurobiological basis of impaired inhibitory control
Banaschewski et al. (9)	2003	Original study	Premonitory sensory phenomena	Early characterization of premonitory urges
Batschelett et al. (19)	2023	Original TMS study	Motor cortex inhibition	Relationship between SICI and tic severity
Brandt et al. (10)	2016	Original study	Temporal relationship between urges and tics	Empirical support for urge–tic coupling
Capriotti et al. (20)	2014	Original study	Negative reinforcement	Tic maintenance through urge relief
Cavanna & Seri (13)	2013	Review	Clinical phenomenology of TS	Reconceptualization of tics as urge-driven behaviors
Conelea & Woods (15)	2008	Review	Contextual modulation of tic expression	Role of environmental and internal factors
Delorme et al. (21)	2016	Original study	Habit formation	Reinforcement and habit-learning mechanisms
Dyke et al. (22)	2022	Systematic review	Non-invasive brain stimulation	Limitations and potential of TMS interventions
Friedrich et al. (23)	2023	Theoretical review	Perception–action integration and CBIT	Behavioral treatment mechanisms
Ganos et al. (7)	2013	Review	Functional anatomy of TS	Neural noise and CSTC dysfunction
Ganos et al. (24)	2014	Neuroimaging study	Tic inhibition	Partial voluntary control and inhibitory mechanisms
Himle et al. (14)	2006	Behavioral review	Tic suppression	Effortful suppression and urge increase
Jackson et al. (11)	2011	Neuroimaging study	Urge-for-action networks	Role of insula and SMA
Johnson et al. (25)	2023	Review	Neurophysiology and treatment	Dynamic sensorimotor dysregulation
Kleimaker et al. (26)	2020	Theoretical paper	Perception–action integration	Coupling between urges and motor output
Larsh et al. (27)	2022	Original TMS study	Cortical excitability and urges	Relationship between urges and cortical modulation
Leckman et al. (8)	1993	Original study	Premonitory urges	Foundational description of PU
Maia & Conceição (5)	2017	Theoretical review	Dopamine and tic learning	Reinforcement-learning framework
Martino & Pringsheim (2)	2018	Review	Clinical and neurobiological overview	Integration of motor and cognitive dysfunction
Münchau et al. (28)	2021	Theoretical review	Neural noise hypothesis	Context-sensitive tic emergence
Orth & Rothwell (16)	2009	Neurophysiology review	Motor cortex excitability	Reduced intracortical inhibition
Piacentini et al. (29)	2010	Randomized clinical trial	CBIT	Behavioral intervention targeting urge–action loop
Rae et al. (30)	2019	Theoretical review	Predictive processing and interoception	Prediction-error account of urges
Robertson (3)	2015	Perspective review	Clinical evolution of TS	Developmental and phenomenological aspects
Rothenberger & Heinrich (31)	2021	Electrophysiology review	Developmental neurophysiology	Compensatory inhibitory processes
Sambrani et al. (32)	2016	Review	Clinical characteristics and PU heterogeneity	Sensory and cognitive dimensions of urges
Schmidgen et al. (33)	2023	TMS/EEG study	State-dependent inhibition	Dynamic inhibitory modulation
Singer (6)	2013	Review	Neurotransmitter systems in TS	Dopaminergic and GABAergic dysfunction
Steuber et al. (34)	2024	Meta-analysis	TMS efficacy in TS	Limited tic reduction but possible urge effects
Tinaz et al. (12)	2015	Neuroimaging study	Sensorimotor cortex involvement	Interoception and motor preparation

Overview of the principal studies included in the present narrative review, including study type, conceptual focus, and contribution to the proposed integrative model of tic generation in Tourette syndrome.

### 2.5 Narrative synthesis

The selected literature was synthesized using a thematic narrative approach. Findings were organized into five domains:

1. neurobiological basis of tics;
2. neurophysiological dynamics;
3. premonitory urges;
4. reinforcement mechanisms;
5. clinical implications.

The aim of the synthesis was not merely descriptive, but integrative. Specifically, the literature was used to develop a model in which tics are conceptualized as regulatory behaviors emerging from dysfunctional neural systems and maintained through reinforcement mechanisms.

### 2.6 Quality and reporting considerations

Although no universal reporting standard exists for narrative reviews, the manuscript was developed according to commonly accepted principles of quality and transparency, including clarity of aims, explicit search strategy, structured synthesis, and theoretical contribution. These principles are consistent with the SANRA framework for narrative reviews (17).

## 3 Results

### 3.1 Neurobiological basis of tics

The neurobiological basis of tics in Tourette syndrome (TS) has been extensively investigated, with converging evidence pointing to dysfunction within CSTC circuits, which are essential for motor control, action selection, and inhibitory processes (4, 7). These circuits consist of reciprocal connections between frontal cortical regions, the basal ganglia, and the thalamus, forming functional loops that regulate the initiation and suppression of motor programs.

Within this framework, tics have been conceptualized as the result of impaired inhibitory gating mechanisms, leading to the inappropriate release of motor patterns. In particular, dysfunction within the striatum is thought to reduce the capacity to suppress competing motor programs, allowing unwanted movements to reach execution threshold (5). This interpretation aligns with classical models of basal ganglia function, which emphasize their role in selecting relevant actions while inhibiting competing ones.

At the neurochemical level, TS has traditionally been associated with dopaminergic dysregulation, supported by the effectiveness of dopamine receptor antagonists in reducing tic severity. However, contemporary models emphasize that TS likely reflects a broader imbalance involving multiple neurotransmitter systems rather than isolated dopaminergic dysfunction alone. In particular, reduced GABAergic inhibition has been proposed as a key mechanism contributing to increased neural excitability and impaired inhibitory control within CSTC circuits (6, 7).

At the same time, clinical evidence supporting the efficacy of alpha-2 adrenergic agonists such as clonidine and guanfacine suggests that noradrenergic systems may also contribute to tic modulation, particularly through effects on attention, arousal regulation, and top-down inhibitory control. In addition, the relationship between psychostimulant medications and tic expression appears more complex than previously assumed. Although stimulants were historically thought to exacerbate tics, more recent evidence suggests that tic worsening is not universal and may depend on individual neurodevelopmental and neurochemical profiles, especially in patients with comorbid attention-deficit/hyperactivity disorder. Together, these findings support the view that tic generation reflects dysregulation across interacting neurotransmitter systems rather than dysfunction within a single neurochemical pathway.

An emerging concept in the neurobiology of TS is that of increased neural noise within motor control systems. According to this perspective, reduced inhibitory precision and abnormal synaptic activity lead to increased background neural activity, thereby increasing the probability that motor patterns are erroneously activated and expressed as tics (7). This framework helps explain both the variability and context sensitivity of tic expression.

Neuroimaging studies further support the involvement of distributed brain networks, including alterations in the basal ganglia—particularly the caudate nucleus—and frontal cortical regions implicated in executive control (2, 7). These findings suggest that tic generation cannot be fully understood as a purely motor phenomenon but rather reflects dysfunction in systems integrating motor, cognitive, and emotional processes.

Taken together, current evidence indicates that the neurobiological basis of tics involves multilevel dysfunction, including impaired inhibitory control within CSTC circuits, neurotransmitter imbalance, and increased neural noise. These mechanisms interact to produce a state of reduced motor stability, in which internally or externally triggered signals are more likely to result in the execution of tic behaviors.

These neurobiological alterations likely influence not only motor output itself, but also the neurophysiological dynamics through which internal states are translated into action. In this sense, abnormalities in inhibitory control and neural excitability may provide the physiological substrate linking dysfunctional neural circuits to the emergence of premonitory urges and tic execution.

### 3.2 Neurophysiological dynamics

Neurophysiological studies suggest that TS is characterized not simply by excessive motor output, but by a broader alteration in the temporal dynamics of sensorimotor and inhibitory processing. Electrophysiological methods, including EEG, event-related potentials (ERPs), TMS, and combined TMS/EEG paradigms, have increasingly supported the view that tic generation reflects abnormal interactions between cortical excitability, motor preparation, perception–action coupling, and compensatory control mechanisms rather than a fixed deficit in a single node of the motor system (25, 31).

A consistent finding across the neurophysiology literature is altered motor inhibition, although the nature of this abnormality appears developmentally and contextually dependent. In children and adolescents, electrophysiological evidence has been interpreted as compatible with a maturational delay of motor inhibition accompanied by cognitive compensation, rather than with a simple, uniform loss of inhibitory capacity. Rothenberger and Heinrich (31), reviewing EEG and ERP findings in younger patients, concluded that TS is associated with cortico-spinal hyperexcitability, immature inhibitory control, and compensatory top-down processes that may help suppress or regulate tic expression in some contexts.

More recent TMS-based work has refined this picture. In a 2023 study of children with TS, reduced short-interval intracortical inhibition (SICI) in primary motor cortex was associated with greater tic severity, while not correlating with urge severity, suggesting that motor cortical inhibition may be more closely linked to tic expression than to the subjective experience of premonitory urges (19). In parallel, Larsh et al. (27) reported that urge severity correlated with altered cortical excitability and long-interval intracortical inhibition, and that SMA GABA-related modulation of motor cortex physiology may reflect compensatory mechanisms in response to urges rather than a purely pathogenic process. Together, these data suggest that TS neurophysiology may involve a dynamic mixture of deficient inhibition and adaptive compensation.

Combined TMS/EEG evidence further indicates that inhibitory physiology in TS is highly state-dependent. Schmidgen et al. (33) found that the TMS-evoked N100, an indirect marker related to GABA_B-mediated cortical inhibition, showed abnormal modulation across different stimulation intensities and motor states in adolescents with TS. Their results were interpreted as suggesting a reduced inhibitory “reserve” at rest and altered regulation during movement preparation and execution. Importantly, this pattern was more compatible with abnormal modulation of excitability than with a stable baseline inhibitory deficit alone, reinforcing the idea that tic generation is embedded in abnormal motor-state transitions.

Another influential line of interpretation conceptualizes TS as a disorder of perception–action integration. Rather than viewing tics as isolated motor discharges, this account emphasizes abnormally strong binding between internal perceptual states, such as premonitory urges, and motor responses. Kleimaker et al. (26) argued that TS is better understood within a theory-of-event-coding framework, in which urges and tics become tightly coupled perception–action units. This perspective is highly relevant neurophysiologically because it implies that abnormalities should be sought not only in motor output but also in the timing and integration of sensory, attentional, and executive signals that precede action. Friedrich et al. (23) extended this logic to behavioral treatment mechanisms, proposing that therapies such as Comprehensive Behavioral Intervention for Tics (CBIT) may act, at least in part, by modifying maladaptive perception–action binding and response selection processes.

A complementary and increasingly important framework is the neural noise model of TS. Münchau et al. (28) proposed that tics, urges, and aspects of cognitive style in TS may be understood in terms of altered signal-to-noise processing across neural systems. In this view, abnormal background activity and reduced precision of information processing increase the likelihood that motor patterns are released inappropriately. This model is appealing because it can integrate findings from scalp EEG, intracranial recordings, and behavioral variability, while also explaining why tic expression is context-sensitive and fluctuating rather than purely stereotyped. It also aligns with the clinical observation that tics may emerge more readily when attentional or inhibitory resources are taxed.

From a translational perspective, neurophysiological findings have also informed neuromodulation studies investigating the potential role of non-invasive brain stimulation in TS. A systematic review of these approaches suggested that repeated stimulation over the SMA may reduce tic severity in some patients; however, the available evidence remains limited by small sample sizes, heterogeneous stimulation protocols, variable outcome measures, and inconsistent replication across studies (22).

More recently, a meta-analysis of randomized sham-controlled TMS studies reported that TMS did not significantly reduce tic severity overall, although a moderate reduction in premonitory urge severity was observed (34). These findings are theoretically interesting within models emphasizing urge-related mechanisms, but they should be interpreted cautiously given the relatively sparse evidence base and methodological variability of the included studies.

Taken together, current TMS findings provide preliminary support for the involvement of motor and interoceptive networks in tic generation, but do not yet establish neuromodulation as a robust or consistently effective therapeutic intervention for TS. Further large-scale and methodologically standardized studies will be necessary to clarify the clinical utility and mechanistic implications of these approaches.

Taken together, current evidence supports the view that the neurophysiological dynamics of TS involve: (a) altered cortical inhibition, especially in motor networks; (b) abnormal modulation of excitability across motor states; (c) enhanced perception–action binding linking urges to tic execution; and (d) possible increases in neural noise that reduce the precision of sensorimotor control. Rather than indicating a single fixed physiological lesion, these findings point to a dynamic dysregulation of sensorimotor control systems, in which tic expression emerges from the interaction between impaired inhibition, altered state regulation, and compensatory processes.

Importantly, these neurophysiological abnormalities are unlikely to operate independently from subjective experience. Altered inhibitory dynamics and perception–action coupling may contribute directly to the emergence of premonitory urges, reinforcing the functional relationship between internal sensory states and tic expression.

### 3.3 Premonitory urges: phenomenology and mechanisms

PU represent one of the most distinctive and clinically relevant features of TS, and are increasingly considered central to its pathophysiology rather than epiphenomenal. These urges are typically described as uncomfortable sensory or cognitive experiences—such as tension, pressure, itching, or a sense of incompleteness—that precede tic execution and are temporarily relieved by it (8, 9).

From a phenomenological perspective, premonitory urges are reported by the majority of adolescents and adults with TS, although their recognition appears to increase with age and cognitive development (32). This developmental trajectory suggests that urges may be present even in younger children but remain less accessible to introspection or verbal report, supporting the notion that they represent a core component of the disorder rather than a secondary phenomenon.

A key aspect of PU is their aversive and tension-like quality, which drives tic execution through a negative reinforcement mechanism. Patients frequently report that performing a tic reduces or temporarily eliminates the urge, reinforcing the behavior over time (20). This urge–tic–relief cycle has led to the conceptualization of tics as behaviors aimed at regulating internal states, rather than purely involuntary motor outputs.

Neurobiologically, premonitory urges have been associated with brain regions involved in interoception and sensorimotor integration, particularly the insula, anterior cingulate cortex (ACC), and SMA. Functional neuroimaging studies suggest that these regions are activated prior to tic execution, supporting the idea that urges represent internally generated signals that trigger motor responses (11, 12). The insula, in particular, is thought to play a key role in representing internal bodily states, while the SMA is implicated in motor preparation and the initiation of voluntary action.

This has led to the interpretation of premonitory urges as a form of abnormal interoceptive signal, arising from altered processing of internal bodily sensations. Rae et al. (30) proposed that TS may involve dysfunction in interoceptive awareness and prediction, resulting in amplified or dysregulated bodily signals that require behavioral discharge through tic expression. Within this framework, tics can be seen as actions that restore homeostatic balance, at least temporarily.

Importantly, the relationship between urges and tics is not purely reflexive but involves elements of volitional control and awareness. Many patients report that tics are performed in response to urges in a way that is experienced as partially voluntary or “unvoluntary,” reflecting a hybrid state between voluntary and involuntary action (24). This intermediate status is supported by behavioral studies showing that individuals with TS can suppress tics for limited periods, although this suppression is typically associated with increasing urge intensity (14).

A study has also highlighted the heterogeneity of premonitory urges, suggesting that they may include both sensory phenomena (e.g., localized bodily sensations) and more abstract experiences such as “just-right” perceptions or feelings of incompleteness (32). This variability further supports the idea that PU reflect a broader disturbance in the integration of sensory, cognitive, and emotional signals.

From a mechanistic perspective, premonitory urges may be understood within a predictive processing framework, in which abnormal prediction errors related to internal bodily states generate persistent signals that drive action. In this view, tics serve to minimize these prediction errors by producing sensory feedback that temporarily resolves the mismatch between expected and actual bodily states (30). This model provides a unifying account linking interoception, motor control, and reinforcement mechanisms.

Taken together, current evidence supports the conceptualization of premonitory urges as core drivers of tic behavior, reflecting abnormal interoceptive processing and contributing to the urge–action–relief cycle that maintains tic expression. Rather than being secondary to motor dysfunction, urges appear to play a central role in shaping both the subjective experience and behavioral dynamics of TS.

From this perspective, tic behaviors may be understood as actions emerging from the interaction between abnormal interoceptive signals, impaired inhibitory control, and learned behavioral responses aimed at regulating internal discomfort.

### 3.4 Tics as urge-driven actions

The conceptualization of tics as urge-driven actions represents a critical shift from traditional models that frame TS as a purely motor disorder. Increasing evidence suggests that tics are better understood as actions performed in response to internally generated signals—namely premonitory urges—rather than as fully involuntary motor outputs (13, 24).

This perspective is supported by phenomenological reports indicating that individuals with TS often experience tics as responses to an internal need or compulsion, rather than as completely uncontrollable movements. Patients frequently describe a subjective sense of agency over tic execution, albeit limited and effort-dependent, leading to the notion of tics as “unvoluntary” actions, occupying an intermediate position between voluntary and involuntary behavior (24). This hybrid status is further supported by experimental findings demonstrating that tic suppression is possible, although typically associated with a progressive increase in urge intensity and discomfort (14).

Within this framework, tics can be conceptualized as goal-directed behaviors, where the “goal” is the reduction of internal tension or the resolution of an aversive sensory state. This interpretation aligns with models of action selection in which behavior is driven not only by external stimuli but also by internal states and motivational signals. In TS, premonitory urges may act as internal drivers that bias action selection toward tic execution, particularly under conditions of reduced inhibitory control (5).

The urge–tic relationship also closely resembles mechanisms observed in reinforcement learning, particularly those involving negative reinforcement. The temporary relief experienced following tic execution reinforces the behavior, increasing the likelihood of its repetition in similar internal contexts (20). Over time, this process may contribute to the consolidation of tic behaviors as habitual responses to specific internal cues, consistent with models of habit learning involving basal ganglia circuits (5).

Recent theoretical frameworks have further elaborated this perspective by integrating concepts from predictive processing and active inference. According to these models, PU may reflect persistent prediction errors related to internal bodily states, which are resolved through action (30). In this context, tics can be interpreted as actions that minimize these prediction errors by generating sensory feedback that restores alignment between expected and actual bodily states. This account provides a mechanistic explanation for why tic execution leads to transient relief and why urges tend to re-emerge over time.

In parallel, the perception–action integration framework suggests that TS involves abnormally strong coupling between internal perceptual states and motor outputs, leading to the rapid and automatic translation of urges into actions (26). This model helps explain why tics can appear highly stereotyped and context-sensitive, as well as why behavioral interventions that target this coupling such CBIT can be effective in reducing tic frequency (23).

Importantly, conceptualizing tics as urge-driven actions has significant implications for understanding their variability and context dependence. Tic expression is known to fluctuate across situations, often increasing under stress, fatigue, or heightened emotional arousal, and decreasing during focused or engaging activities. This variability is consistent with a model in which tic expression reflects the dynamic interaction between internal drive (urge intensity), inhibitory capacity, and environmental demands.

Taken together, the available evidence supports a model in which tics are not merely the result of motor disinhibition, but rather actions emerging from the interaction between internal urges, impaired inhibitory control, and reinforcement mechanisms. In this view, TS can be understood as a disorder of action regulation, in which internally generated signals disproportionately influence behavior, leading to the recurrent expression of tic behaviors.

### 3.5 Reinforcement mechanisms

A growing body of evidence supports the role of reinforcement learning mechanisms in the maintenance and consolidation of tic behaviors in TS. While early models emphasized motor disinhibition, more recent frameworks highlight how tic expression may be shaped over time through learning processes driven by internal reinforcement signals (5, 20).

Central to this perspective is the concept of negative reinforcement, whereby the execution of a tic leads to a temporary reduction in the intensity of premonitory urges. This relief functions as a reinforcing outcome, increasing the probability that the same behavior will be repeated in response to similar internal states (20). Over repeated cycles, this process may contribute to the stabilization of tic behaviors as conditioned responses to specific interoceptive cues.

This mechanism is consistent with broader models of habit formation, in which behaviors initially driven by goal-directed processes become progressively automatized through repeated reinforcement. In TS, premonitory urges may act as internal cues that trigger learned stimulus–response associations, with tic execution becoming increasingly habitual over time (21). This interpretation aligns with the known involvement of basal ganglia circuits in both tic generation and habit learning.

Importantly, reinforcement processes in TS may not be limited to negative reinforcement alone. In some cases, tic execution may also be associated with sensory satisfaction or “*just-right*” experiences, which could contribute to positive reinforcement mechanisms, particularly in complex or ritualized tics (13). This dual reinforcement model may help explain the persistence and variability of tic behaviors across individuals.

Recent work has also explored reinforcement learning abnormalities in TS using computational approaches. For instance, alterations in reward sensitivity and prediction error processing have been reported, suggesting that individuals with TS may process reinforcement signals differently, potentially contributing to the persistence of maladaptive behaviors (Palminteri et al., 2011; 5). These findings support the idea that tic behaviors are embedded within broader learning systems rather than representing isolated motor phenomena.

From a clinical perspective, the role of reinforcement is particularly relevant for behavioral interventions such as CBIT, which explicitly target the urge–tic relationship and aim to disrupt reinforcement cycles. By introducing competing responses and increasing awareness of urges, these interventions may reduce the reinforcing value of tic execution and promote alternative behavioral patterns (29).

Taken together, these findings support a model in which tic behaviors are maintained through reinforcement loops linking internal urges to motor output, with both negative and, in some cases, positive reinforcement contributing to their persistence. This perspective complements neurobiological models by providing a mechanism through which transient neural dysfunctions may translate into stable behavioral patterns.

## 4 Discussion

### 4.1 Toward an integrative model

The evidence reviewed so far suggests that no single-level explanation is sufficient to account for the complexity of tic generation in TS. Instead, a comprehensive understanding requires the integration of neurobiological, neurophysiological, and psychological mechanisms into a unified framework.

At the neurobiological level, dysfunction within CSTC circuits results in impaired inhibitory control and increased motor system excitability, creating a permissive environment for the emergence of tic behaviors (4, 7). At the neurophysiological level, abnormalities in cortical inhibition, motor preparation, and state-dependent modulation of excitability further contribute to instability in motor output systems (25, 31).

At the psychological level, PU represent internally generated signals that drive behavior, acting as proximal triggers for tic execution (8, 32). These urges are experienced as aversive and are temporarily relieved by tic performance, establishing a functional link between internal states and motor actions.

Crucially, these levels are not independent but interact dynamically within a closed-loop system. In this model, dysfunction in neural circuits gives rise to abnormal interoceptive signals (premonitory urges), which in turn trigger motor responses (tics). The execution of tics produces temporary relief, reinforcing the behavior through negative reinforcement mechanisms and increasing the likelihood of future tic expression under similar conditions.

This loop can be summarized as:


Premonitory urge → Tic execution → Relief → Reinforcement → Increased probability of future tic.

**Figure 2:**
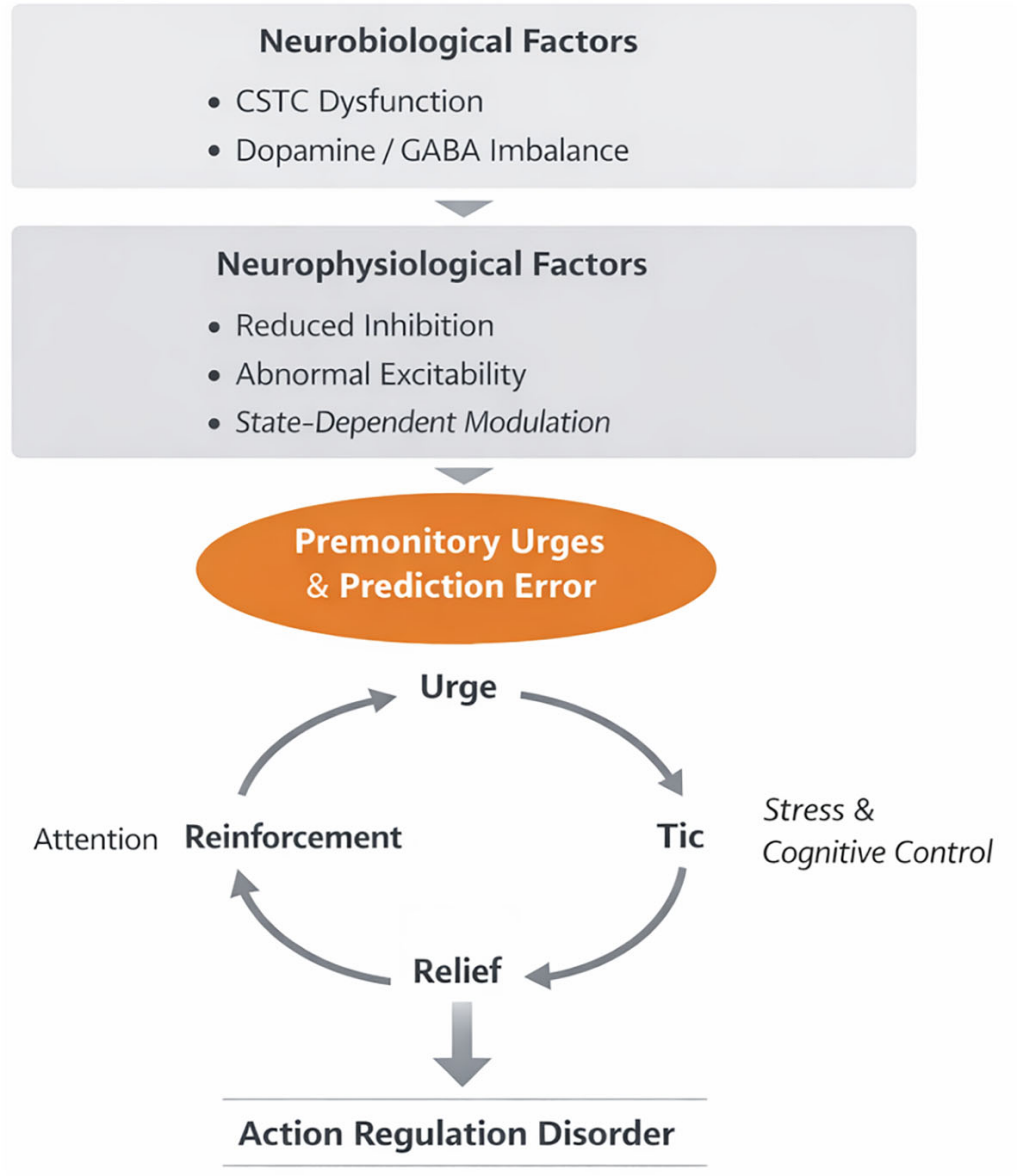
Integrative model of tic generation in Tourette syndrome. A multilevel conceptual framework illustrating the interaction between neurobiological, neurophysiological, and psychological mechanisms involved in tic generation in Tourette syndrome. Dysfunction within cortico–striato–thalamo–cortical circuits, altered inhibitory control, and abnormal cortical excitability contribute to the emergence of premonitory urges, conceptualized as abnormal interoceptive signals and prediction-related processes. These urges act as proximal drivers of tic execution within a dynamic urge–action–relief loop. Tic performance produces temporary relief, reinforcing behavior through negative reinforcement mechanisms and increasing the likelihood of future tic expression. Modulatory factors such as attention, stress, and cognitive control influence the intensity and expression of tic behaviors. The model conceptualizes Tourette syndrome as a disorder of action regulation emerging from the interaction between dysfunctional neural systems, internal bodily signals, and reinforcement-based learning processes.

This integrative framework is illustrated in **Figure 2**.

Despite the integrative value of the proposed framework, alternative conceptualizations of tic generation should also be acknowledged. Traditional models have primarily interpreted tics as the direct consequence of motor disinhibition resulting from dysfunction within cortico–striato–thalamo–cortical circuits (4). Other perspectives have emphasized habit-learning mechanisms and reinforcement processes as the primary drivers of tic persistence (5), while more recent predictive processing accounts conceptualize tics as actions aimed at minimizing interoceptive prediction errors (30).

Importantly, significant empirical uncertainty remains regarding the precise causal relationship between premonitory urges and tic execution. Although urges are commonly experienced prior to tics and appear functionally linked to them, it is still unclear whether premonitory urges represent primary triggers of tic generation or secondary phenomena emerging from underlying neural dysfunction (24). It is also possible that urges and tics arise from partially overlapping but dissociable neurophysiological processes. The present framework should therefore be understood not as a definitive account of tic generation, but as a working integrative model intended to organize current evidence and generate empirically testable hypotheses.

Importantly, this model also accommodates the role of perception–action coupling and neural noise hypotheses. From this perspective, tics may emerge when internally generated sensorimotor signals exceed the threshold for motor execution under conditions of reduced inhibitory filtering and increased neural variability (26, 28). In this context, the concept of “neural noise” refers to a reduced signal-to-noise ratio within motor and sensorimotor networks, increasing the probability that internally generated urges or motor representations are translated into overt action. Rather than reflecting a single abnormal movement pathway, tic generation may therefore arise from dynamic fluctuations in excitability, inhibition, attention, and contextual modulation, contributing to the characteristic variability and context sensitivity of tic expression.

Another key feature of this integrative model is the presence of compensatory mechanisms. Individuals with TS often develop strategies to suppress or modulate tics, particularly in socially demanding contexts. Neurophysiological and behavioral evidence suggests that these compensatory processes involve increased recruitment of cognitive control systems, which may partially counterbalance underlying neural dysfunction (31).

A developmental perspective is also essential for understanding the evolution of tic disorders over time. Tic severity often decreases from adolescence into adulthood, suggesting that maturational processes may progressively improve inhibitory control and sensorimotor regulation (3). Neurodevelopmental models propose that the maturation of frontal control systems and compensatory neural mechanisms may partially counterbalance the underlying dysfunction of CSTC circuits, contributing to symptom reduction in many individuals with TS (31). At the same time, the persistence of clinically significant tics in a subset of patients suggests that these compensatory processes may vary substantially across individuals. Integrating developmental dynamics into the present framework may therefore help explain both the heterogeneity and longitudinal variability of tic expression.

This integrative perspective has important clinical implications. It suggests that effective interventions should target not only motor symptoms but also the underlying urge–action–reinforcement loop, for example by reducing urge intensity, enhancing inhibitory control, or disrupting learned associations between urges and tic execution.

In summary, TS can be conceptualized as a disorder of action regulation, in which tics emerge from the interaction between dysfunctional neural systems, abnormal interoceptive signals, and reinforcement-based learning processes. This framework provides a unifying account that bridges multiple levels of analysis and may offer a foundation for more targeted and effective therapeutic approaches.

### 4.2 Empirical testability of the model

A central implication of the proposed framework is that its components can be empirically tested across multiple levels of analysis. First, the temporal relationship between premonitory urges and tic execution could be examined using ecological momentary assessment, wearable sensors, and real-time tic monitoring, approaches that may help clarify whether fluctuations in urge intensity reliably predict tic occurrence in naturalistic settings (10, 20).

Second, the relationship between interoceptive signals and motor output could be investigated through paradigms combining urge ratings with EEG, TMS, or functional neuroimaging during tic suppression and tic release. Such approaches may help determine whether altered cortical inhibition and motor excitability mediate the transition from urge to action (19, 33).

Third, the reinforcement component of the model could be explored by measuring changes in urge intensity before and after tic execution, as well as by examining whether the magnitude of relief predicts future tic frequency or resistance to suppression, consistent with negative reinforcement accounts of tic maintenance (20).

Finally, clinical trials could assess whether interventions specifically targeting urge awareness, competing responses, interoceptive tolerance, or inhibitory control produce changes not only in tic severity, but also in the strength of the urge–action–relief association. In particular, behavioral approaches such as Comprehensive Behavioral Intervention for Tics (CBIT) may provide an ideal framework for testing whether modifying perception–action coupling reduces tic expression over time (23, 29).

Taken together, these approaches suggest that the proposed framework generates empirically testable and potentially falsifiable predictions linking neurophysiological markers, subjective urge experience, behavioral expression, and treatment response.

### 4.3 Clinical implications

The proposed framework may also have important implications for clinical intervention in Tourette syndrome. Conceptualizing tics as urge-driven regulatory behaviors suggests that treatment should not focus exclusively on suppressing motor symptoms, but also on modifying the relationship between internal urges, action selection, and reinforcement processes.

Behavioral interventions such as Comprehensive Behavioral Intervention for Tics (CBIT) are particularly consistent with this perspective, as they target the urge–action relationship through awareness training, competing responses, and modification of reinforcement contingencies (23, 29). Within the present model, these interventions may reduce tic expression not only by increasing behavioral control, but also by weakening maladaptive perception–action coupling and reducing the reinforcing value of tic execution.

The framework also suggests that interventions aimed at improving interoceptive tolerance and inhibitory control may represent promising therapeutic directions. For example, mindfulness-based approaches, urge-monitoring strategies, and neuromodulatory interventions targeting motor and interoceptive networks could potentially reduce the intensity of premonitory urges or alter the transition from urge to action (22).

Importantly, the model supports a more individualized understanding of tic disorders, in which treatment may need to be adapted according to differences in urge intensity, inhibitory capacity, reinforcement sensitivity, and developmental stage. This perspective may help explain the heterogeneity of treatment response observed in clinical practice and supports the development of more personalized interventions targeting the mechanisms underlying tic generation rather than tic frequency alone.

## 5 Limitations

Despite the integrative scope of the present review, several limitations should be acknowledged.

First, the narrative design of this work, while appropriate for theory development, does not allow for quantitative synthesis or formal estimation of effect sizes. As such, the proposed model is based on a qualitative integration of heterogeneous findings rather than on meta-analytic evidence. Although this approach enables conceptual advancement, it may introduce a degree of interpretative bias in the selection and weighting of the literature (17).

Second, the available empirical literature remains methodologically heterogeneous. Studies included in this review differ substantially in terms of population characteristics (e.g., age, tic severity, comorbidities), experimental paradigms, and neurophysiological measures. This heterogeneity limits the ability to draw definitive conclusions regarding causal mechanisms and may reduce the generalizability of the proposed framework.

Third, while the model emphasizes the central role of premonitory urges and reinforcement processes, the precise causal relationship between urges, neural activity, and tic execution remains incompletely understood. In particular, it is still unclear whether premonitory urges represent a primary driver of tic generation or a secondary phenomenon emerging from underlying neural dysfunction. Future studies employing longitudinal and experimental designs will be necessary to disentangle these relationships.

Fourth, although recent neurophysiological and computational frameworks (e.g., predictive processing, reinforcement learning) offer promising explanatory models, their application to Tourette syndrome remains largely theoretical. Empirical validation of these models is still limited, and further work is needed to test their predictions using multimodal approaches combining behavioral, electrophysiological, and neuroimaging data.

In addition, although the present framework integrates neurobiological and neurophysiological findings, it does not comprehensively address genetic and pharmacological contributions to tic generation. Future models may benefit from incorporating these domains in order to achieve a more fully multidimensional understanding of Tourette syndrome.

Finally, the present model does not fully account for the influence of developmental factors and comorbid conditions, such as attention-deficit/hyperactivity disorder and obsessive–compulsive disorder, which are highly prevalent in Tourette syndrome and may significantly modulate both urge experience and tic expression. Future research should aim to incorporate these dimensions into more comprehensive and individualized models of tic generation.

## 6 Conclusion

TS can no longer be fully understood as a purely motor disorder. The evidence reviewed in this paper supports a reconceptualization of tics as urge-driven, regulatory behaviors emerging from the interaction between dysfunctional neural circuits, abnormal interoceptive signals, and reinforcement-based learning processes.

Within this framework, premonitory urges represent central drivers of tic expression, linking internal states to motor output through a dynamic urge–action–relief loop. Neurobiological and neurophysiological alterations create a permissive context for tic generation, while reinforcement mechanisms contribute to their persistence over time.

By integrating findings across multiple levels of analysis, the present model provides a unified account of tic generation and highlights Tourette syndrome as a disorder of action regulation. This perspective not only advances theoretical understanding but also has potential implications for clinical assessment and intervention, emphasizing the importance of targeting the functional relationship between urges and behavior.

Future research should aim to empirically test this integrative framework and further clarify the mechanisms linking interoception, inhibition, and learning processes in tic disorders.

## Data Availability

The raw data supporting the conclusions of this article will be made available by the authors, without undue reservation.
